# Tolerant indirect reciprocity can boost social welfare through solidarity with unconditional cooperators in private monitoring

**DOI:** 10.1038/s41598-017-09935-2

**Published:** 2017-08-29

**Authors:** Isamu Okada, Tatsuya Sasaki, Yutaka Nakai

**Affiliations:** 1 Soka University, Faculty of Business Administration, Tokyo, 192-8577 Japan; 2 University of Vienna, Faculty of Mathematics, Vienna, 1090 Austria; 3 Shibaura Institute of Technology, Faculty of Systems Engineering and Science, Saitama, 337-8570 Japan

## Abstract

Indirect reciprocity is an important mechanism for resolving social dilemmas. Previous studies explore several types of assessment rules that are evolutionarily stable for keeping cooperation regimes. However, little is known about the effects of private information on social systems. Most indirect reciprocity studies assume public monitoring in which individuals share a single assessment for each individual. Here, we consider a private monitoring system that loosens such an unnatural assumption. We explore the stable norms in the private system using an individual-based simulation. We have three main findings. First, narrow and unstable cooperation: cooperation in private monitoring becomes unstable and the restricted norms cannot maintain cooperative regimes while they can in public monitoring. Second, stable coexistence of discriminators and unconditional cooperators: under private monitoring, unconditional cooperation can play a role in keeping a high level of cooperation in tolerant norm situations. Finally, Pareto improvement: private monitoring can achieve a higher cooperation rate than does public monitoring.

## Introduction

The eyes of others make people act morally. Even subtle surveillance cues can influence cooperative behavior as shown in a series of social psychological experiments^1–3^. Why does surveillance make people cooperative? One explanation from the evolutionary perspective is that the doers anticipate how the observers assess the doers’ behaviors^4–6^. A person’s moral code may depend on the type of monitoring^7^. The adaptive moral code in a weak monitoring system, in which few of the potential observers can observe the behaviors, may differ from that in a strong monitoring system, in which most of the potential observers can observe the behaviors. A new question then arises. Does the type of monitoring enhance the cooperative behaviors?

**Figure 1:**
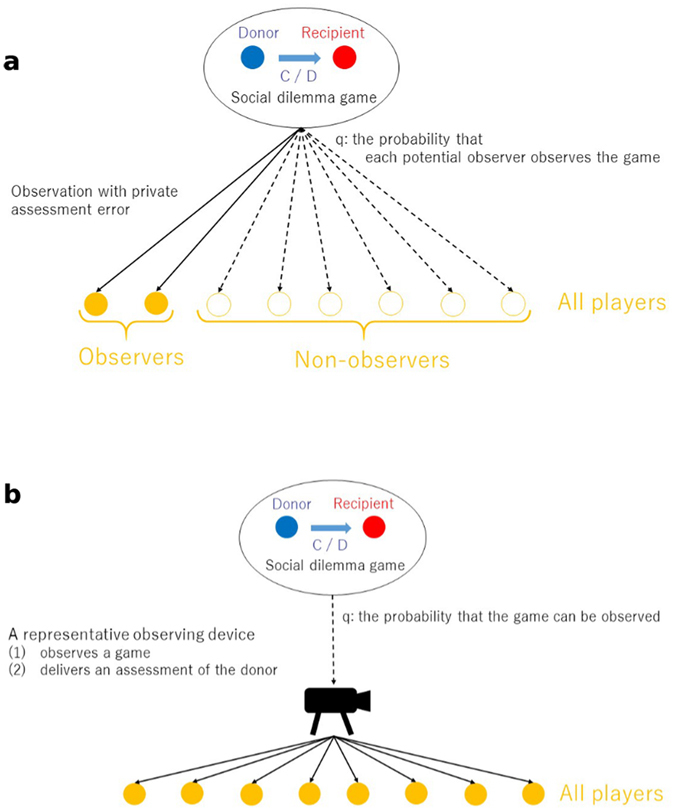
Two monitoring systems in indirect reciprocity. (**a**) Private monitoring: each potential observer can observe a game independently with the probability, *q*, and otherwise cannot. The actual observers privately assess a donor of the game and the non-observers never update their assessments of the donor. (**b**) Public monitoring: a representative device observes a game and delivers its public assessment of a donor in the game to all of players with the probability, *q*; otherwise, the device does not observe the game and the assessment of any player is never updated.

To address the question, we analyze indirect reciprocity in two monitoring systems: private and public (Fig. 1). We consider indirect reciprocity because assessment rules and moral judgment have been theoretically^8–14^ and empirically^15–20^ considered in studies on the evolution of cooperation by indirect reciprocity. Those studies show that assessment rules realizing retributive justice–to help those who help the good and to not help those who do not help the good–are necessary to evolutionarily stabilize a cooperative regime. Retributive justice has a long history, ranging from Moses’s Revenge (Deuteronomy 7:1–2) and the Code of Hammurabi, to Immanuel Kant (*the law of talion: the punishment corresponds in kind and degree to the injury*)^21^ and Georg Hegel^22^ (*if the crime is a denial of the rightness of the rule broken, then the negation of that denial restores the rule*) [Ref. 23, p. 48], which suggest that whoever takes another’s life should pay the ultimate price. We comparatively investigate some retributive assessment rules in private and public monitoring systems.

The “image-scoring”^24, 25^ norm is a pioneering solution of indirect reciprocity in the evolution of cooperation. Discriminators who adopt image-scoring, tag cooperators as *good* and non-cooperators (“defectors”) as *bad*, and help the *good* players only. However, the norm is vulnerable when errors in both implementation and perception occur and when mutations arise^26^. This is because a norm adopter’s defection to a *bad* player simultaneously hurts her or his own reputation with the other adopters of the norm, and thus, a defecting norm adopter becomes the next *bad* player. Therefore, the chain of those with a *bad* reputation infinitely continues and the cooperative regime cannot remain.

The key point in solving this vulnerability is discriminating between a justified defection performed by the discriminators and an unjustified defection performed by the all-out defectors. One notable solution is to consider second-order information^27^. The image-scoring rule relies only on information about the donor’s action, which is first-order information^28^. A defection by a discriminator has a justified reason, such as: “I refuse to help because the recipient has a *bad* reputation”. Then, to discriminate whether the defection is justified or unjustified, the discriminator needs to consider the recipient’s reputation, which is the second-order information. If the discriminators use both first- and second-order information, then justified defection including punishment works.

Theoreticians have discovered several norms using the second- and/or higher-order information that are evolutionarily stable in cooperative regimes, even with errors. Ohtsuki and Iwasa’s detailed analysis^29^ shows that the eight assessment rules are evolutionarily stable and can achieve substantially high cooperation levels. The “leading eight” norms have a common feature of their assessment rules: cooperation for a *good* recipient is assessed as *good* and defection for a *good* recipient is assessed as *bad*. There are several different norms for a *bad* recipient. The most tolerant norm, which is called “simple-standing”^30^, is that both cooperation and defection for a *bad* recipient are assessed as *good*; and the stricter norm, which is called “stern-judging”^31^, is that cooperation for a *bad* recipient is assessed as *bad* while defection is *good*. The strictest norm, which is not in the leading eight, is called “shunning”^32^ where any action for a *bad* recipient is assessed as *bad*. Shunning is robust for the invasion of unconditional cooperators and defectors, yet can lead to reducing the level of cooperation in the long run^33^. Those four norms (image-scoring, simple-standing, stern-judging and shunning) are regarded as the most popular social norms^34^. Additionally, a new norm that shares the common feature is called “staying”^35–37^, which has been proved to be as stable as are the leading eight norms. In the staying norm, the image of the potential donor remains unchanged if the potential recipient has a *bad* image.

Most theoretical studies on indirect reciprocity assume *public* monitoring in which all observation is public and shared^38, 39^ (Fig. 1b). Decentralized ways of spreading reputations, similarly effective in making reputations public, have frequently been suggested in the literature, with gossiping being the most pressing example^40^. By this assumption, all of the players share the same image of a player even if an error in perception occurs, and thus, each player has a single image that does not vary among the other players. Essentially, society is dominated by an overarching surveillance system that monitors all behaviors and broadcasts its judgments, so that individual residents are not permitted to make a personal assessment of the other residents. This surveillance system incurs assessment and broadcasting costs; hence, the second-order free-riders emerge who shirk the cost burden^41, 42^. Moreover, people naturally have their private assessments of others.

In contrast to public monitoring, in *private* monitoring (Fig. 1a), only some of the potential observers can observe the players’ behaviors. Hence, each observer may have a different image of a player. The private assessment scheme for implementing private monitoring requires an image matrix^35, 36, 43^, that is described by an *N* × *N* square matrix, in which *N* is the number of players and its (*x*, *y*) component denotes an image of player *y* in the eyes of player *x*. In the case of the public information scheme, the image matrix reduces to a vector of dimension *N*. A private monitoring system is difficult to analyze, and only a few studies attempt it^28, 32, 44–47^.

Here, we systemically show the stable norms in the private monitoring system and compare them with those in the public monitoring system. To do so, we use individual-based simulations^48^ by which all of the major norms can be dealt with and private assessment can be implemented without any approximation (see Methods for details). Our main contribution is to clarify that the unconditional cooperators play a role in keeping a high level of cooperation in combination with the norms in the private monitoring system. The trade-off is that cooperative regimes in private monitoring become unstable.

## Results

### Strict norms are unlikely to evolve in private monitoring

Our simulation result shows that the cooperative stable norms are more restricted in private monitoring than those in public monitoring, as shown in Tables 1 and 2. Particularly, this result shows that the strict norms (stern-judging and shunning) are not stable in the private monitoring system while they can be in the public monitoring system^33, 37^.

**Table 1:** The cooperative stable norms in private monitoring.

Norm	Stability	Cooperation rate
Image-scoring	72%	95.87%
Simple-standing	77%	92.57%
Stern-judging	9%	50.57%
Shunning	35%	n/a
Staying	97%	95.20%

The stability results represent the ratio of the trials where the frequency of unconditional defectors is less than halved at the end of 100 generations. The cooperation rate results represent the average of cooperation rates after 100 generations of the trials where those values exceed 0.5. Each case is carried 100 trials. The parameter values are *N* = 100, *b* = 3, *c* = 1, *e*_1_ = 0.03, *e*_2_ = 0.03, *T* = 100,000, *T*_*s*_ = 90,000, *μ* = 0.001, *β* = 3, and *q* = 0.01. An initial population consists of 100% discriminators.

This is because, in a public monitoring system, assessment information is public and is equally shared among all players. In stern-judging, every justified defection by a discriminator precisely gets the point across to all players just as the discriminator intended (defection for *bad* is assessed as *good*). In shunning, there is a small but certain amount of discriminators who are assessed as *good*. If a discriminator is assessed as *good*, then the discriminator receives a great benefit because all of the discriminators assess the discriminator as *good*. This prevents the unconditional defectors from invading and the homogeneous state of the discriminators is stable.

Contrastingly, a private monitoring system has a very different situation. The absence of public broadcast does not guarantee that the other players share concurrent assessments. In stern-judging, an observer may not precisely understand a donor’s intention of justified defection, and thus, the justified defection may undermine the donor’s own assessment. This downgrade is also seen in shunning because a *good* reputation cannot remain in the homogeneous state of the discriminators, unless a recipient’s images by both a donor and an observer are equally *good* and an error in implementation never occurs. This is why, in both strict norms, unconditional defectors can invade into the norms, and the norms are not stable in a private monitoring system.

### Stable mixture of discriminators and unconditional cooperators

**Figure 2:**
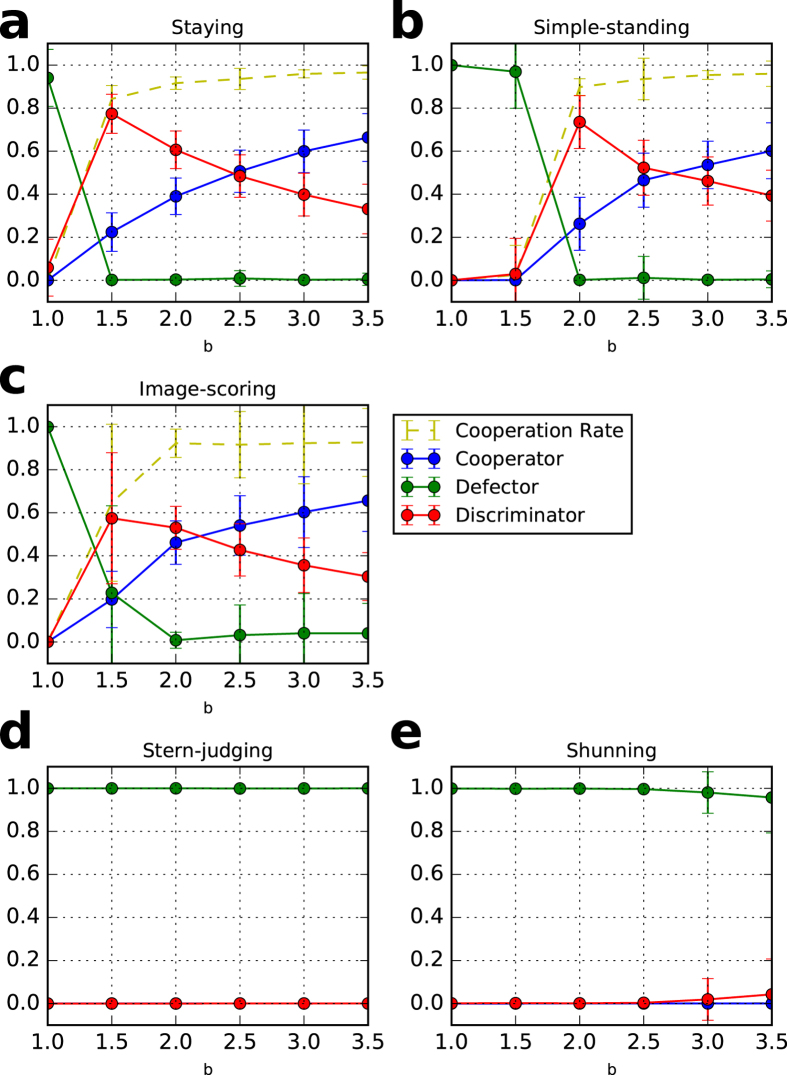
Cooperation rates and strategies in a private monitoring system with different benefits, *b*. Each dot reflects an average of 100 trials after 30 generations with six values of *b*. (**a**) staying, (**b**) simple-standing, (**c**) image-scoring, (**d**) stern-judging, and (**e**) shunning. The error bars represent one standard deviation of the data. In the initial population, 10% are unconditional cooperators, 10% are unconditional defectors, and 80% are discriminators. The parameter values are *N* = 100, *q* = 0.01, *c* = 1, *e*_1_ = 0.03, *e*_2_ = 0.03, *T* = 100,000, *T*_*s*_ = 90,000, *μ* = 0.001, and *β* = 3.

**Figure 3:**
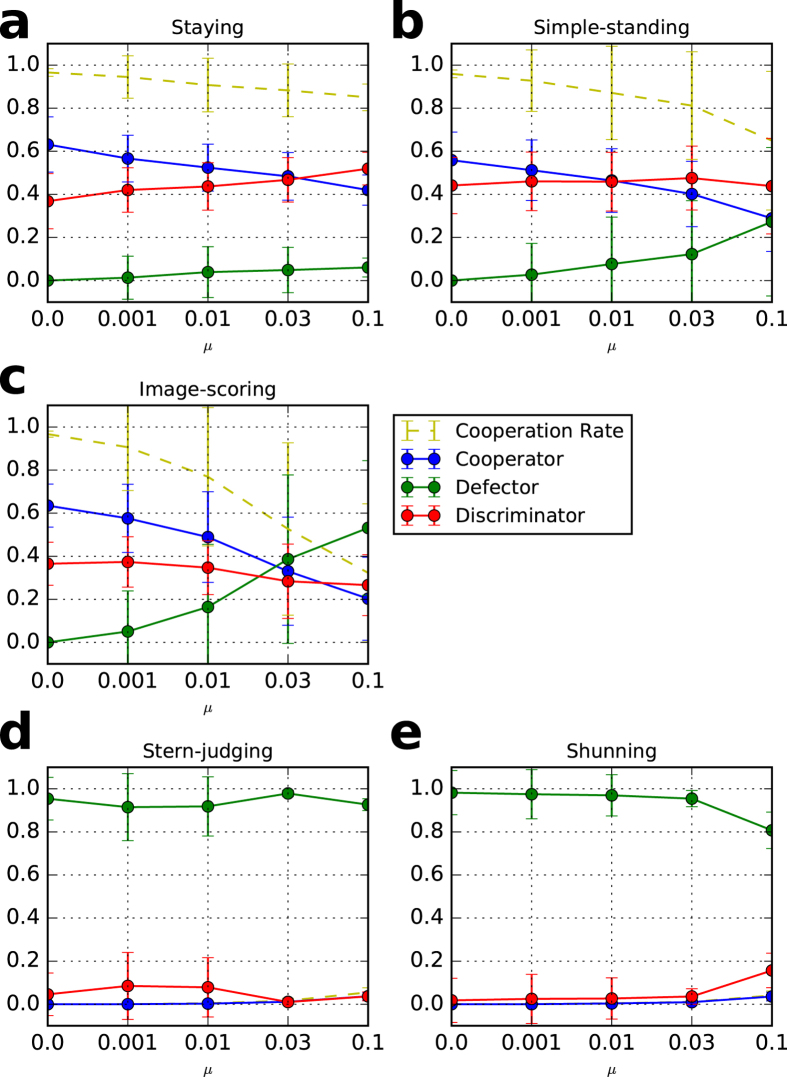
Cooperation rates and strategies in a private monitoring system with different mutation rate, *μ*. Each dot reflects an average of 100 trials after 30 generations with five values of *μ*. (**a**) staying, (**b**) simple-standing, (**c**) image-scoring, (**d**) stern-judging, and (**e)** shunning. The error bars represent one standard deviation of the data. In the initial population, 10% are unconditional cooperators, 10% are unconditional defectors, and 80% are discriminators. The parameter values are *N* = 100, *q* = 0.01, *b* = 3, *c* = 1, *e*_1_ = 0.03, *e*_2_ = 0.03, *T* = 100,000, *T*_*s*_ = 90,000, and *β* = 3.

The second feature of the private monitoring system is that two of the cooperative stable norms (simple-standing and staying) are more likely to coexist with unconditional cooperators in contrast with in the public monitoring system, as shown in Figs 2 and 3. In a public monitoring system, simple-standing and staying neither act deviantly nor hurt their reputations when the system has no errors. This is because, in simple-standing, whenever the image of the recipient in the eyes of the donors is *bad*, that in the eyes of the observers is absolutely *bad*, and thus, the observers precisely understand the donor’s justified defection.

Contrastingly, in a private monitoring system, the images of the recipient in the eyes of the donor and of the observers may not be the same, and thus, any discriminator is assessed as *bad* by even a small number of the other discriminators. The *bad* reputation erases an advantage of the discriminators over the unconditional cooperators because the unconditional cooperators do not downgrade their assessments because they never defect. Therefore, the unconditional cooperators can invade into the homogeneous state of the discriminators, which they cannot do in a public monitoring system. Because the appropriate mixture of discriminators and unconditional cooperators economically defend against an invasion of defectors, the mixture is stable.

While image-scoring seems to have a similar result, it cannot be stable because the system is vulnerable due to the large deviation shown in Figs 2 and 3c. This vulnerability is supported by a theoretical analysis using a replicator dynamics by Sigmund^26^ that shows that image-scoring and unconditional cooperators neutrally drift.

**Figure 4:**
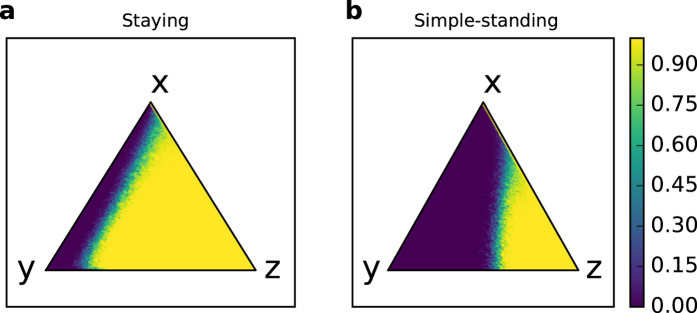
Basin of a cooperative regime in the private monitoring system. (**a**) staying and (**b**) simple-standing. The triangles describe a simplex of the state space, *S* = {(*x*, *y*, *z*): *x* + *y* + *z* = *N*}, where *x*, *y*, and *z* are non-negative integers denoting the frequencies of unconditional cooperators, unconditional defectors, and discriminators, respectively. The colored dots correspond to the probability of finally reaching a cooperative regime. Here, ten trials were performed on each point, (*x*, *y*, *z*) ∈ *S*. The borders of the basin of attraction are approximately (**a)** *z* = 0.15 and (**b)** *z* = 0.4. The averages of the probabilities for all of the points to finally reach a cooperative regime are (**a)** 76.04% and (**b**) 33.37%. The parameter values are *N* = 100, *b* = 3, *c* = 1, *e*_1_ = 0.03, *e*_2_ = 0.03, *T* = 100,000, *T*_*s*_ = 90,000, *μ* = 0, and *β* = 3.

Figure 4 shows the basins of attraction for the stable coexistence of discriminators and the unconditional cooperators in staying and simple-standing, respectively. As shown in the figure, the basin of attraction for the cooperative stable point in staying is wider than that in simple-standing. Basically, the basins depend on the fraction of the discriminators exceeding a threshold. To investigate the basin of attraction for the cooperate regime we take a stochastic process approach. Using individual-based simulations, we calculate a state transition matrix. See the Methods section for details of calculating the basin of attraction in the model.

### Unstable cooperation

**Figure 5:**
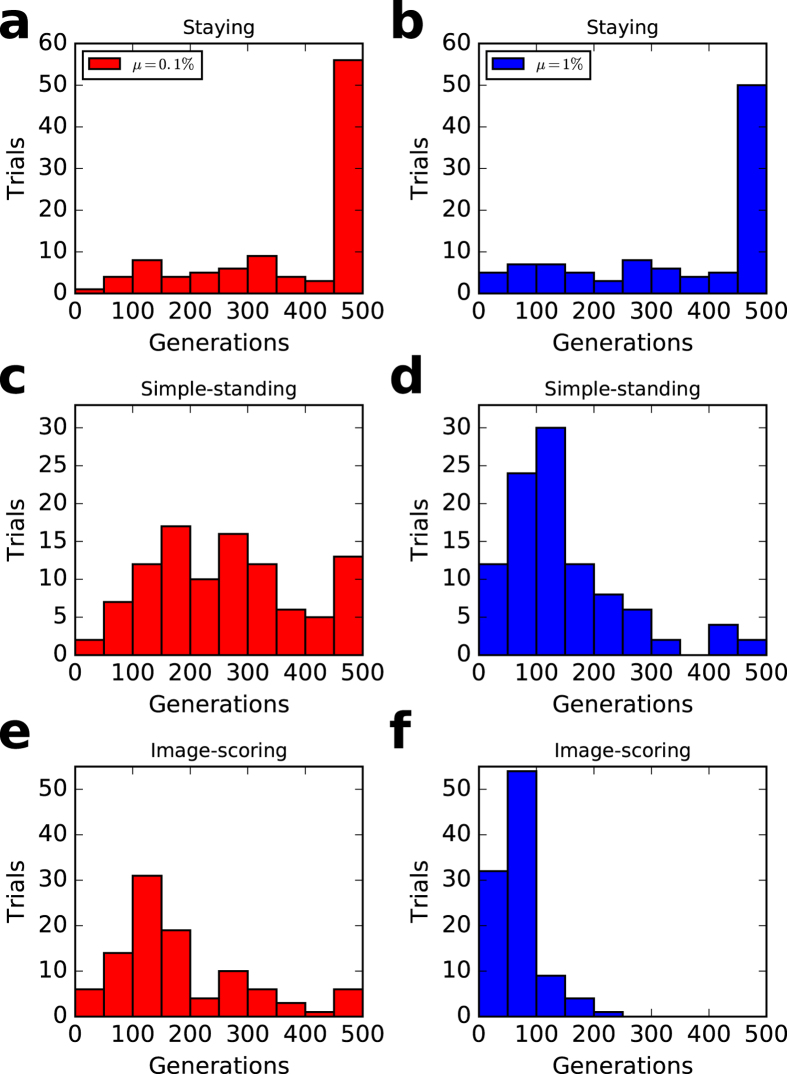
Generation lengths for keeping cooperative regimes. (**a** and **b**) staying, (**c** and **d**) simple-standing, (**e** and **f**) image-scoring. (left) *μ* = 0.1% and (right) *μ* = 1%. In each trial, a run was broken off if either the cooperation rate was smaller than 0.2 or it reaches 500 generations. The histogram data are the generation lengths of 100 trials in each case. Each initial population consists of 100% discriminators. The parameter values are *N* = 100, *q* = 0.01, *c* = 1, *e*_1_ = 0.03, *e*_2_ = 0.03, *T* = 100,000, *T*_*s*_ = 90,000, and *β* = 3.

In the private monitoring system, even tolerant norms do not necessarily keep cooperative regimes in the long term. As shown in Fig. 5, the generation lengths keeping cooperative regimes are finite, and staying has the longest length, simple-standing the second longest, and image-scoring has the shortest of the three. This is consistent with Table 1 and Fig. 4. Table 1 shows that staying, simple-standing, and image-scoring, respectively, cannot keep cooperative regimes in 3, 23, and 28 of 100 trials. Comparing staying with simple-standing in Fig. 4, the wider the basin of attraction, the more robust the cooperative regime kept.

Why can simulations of the private monitoring version not keep cooperative regimes in the long term? The private monitoring system has cooperative regimes in combination with unconditional cooperators and discriminators, and thus, their payoffs are almost the same and their fractions are highly flexible due to instability intrinsically installed in the simulation. The instability is included in the updating process (Fermi function) and randomness (initialization, matching, and mutation processes). For example, a player receives a higher payoff if the number of playing recipients is greater than the others due to the random matching process. A discriminator must pay a cost if one meets a *good* player. In contrast with simulations in which players use the actual values in an updating process, theoretical analyses may be used with the expected values, and thus, there is no flexibility. We need a rigorous analysis of easily stable states using theoretical analysis for future works.

### Pareto improvement

**Figure 6:**
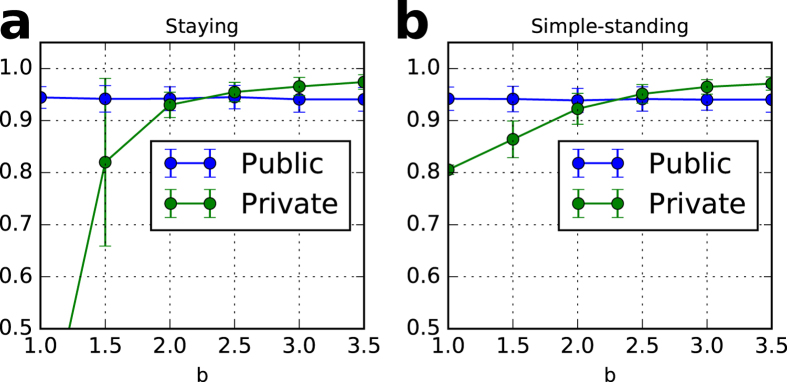
Cooperation rates of stable states with private monitoring and public monitoring. (**a**) staying and (**b**) simple-standing. Each dot reflects an average of 100 trials after 30 generations with six values of benefits, *b*. Each initial population consists of 50% unconditional cooperators and 50% discriminators. The cooperation rate with private monitoring exceeds that with public monitoring if *b* 2.0 in both (**a)** and (**b)**. The parameter values are *N* = 100, *q* = 0.01, *c* = 1, *e*_1_ = 0.03, *e*_2_ = 0.03, *T* = 100,000, *T*_*s*_ = 90,000, *μ* = 0, *β* = 3, and *q* = 0.01 in (**a**) and *q* = 1 in (**b**).

**Figure 7:**
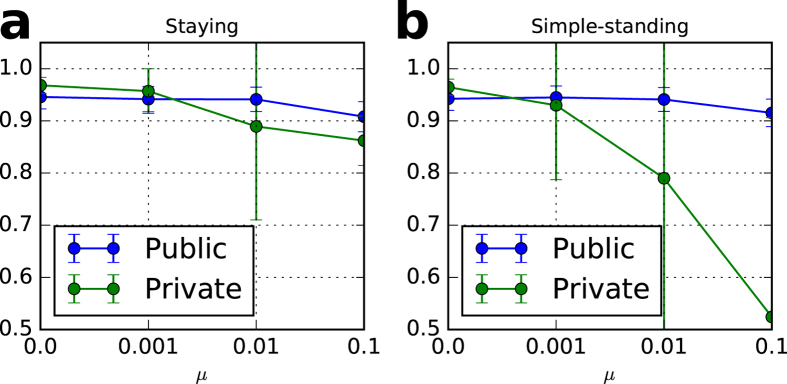
Cooperation rates of stable states with different mutation rate, *μ*. (**a**) staying and (**b**) simple-standing. The cooperation rate with private monitoring exceeds that with public monitoring if *μ* < 0.01 in both (**a** and **b**). The simulation settings are the same as Fig. 6 except for *b* = 3 and *μ* (a variable).

In exchange for unstable cooperation, the private monitoring system improves the Pareto efficiency, as shown in Figs 6 and 7 and Supplementary Information. The cooperation rate of the stable state in the private monitoring system is higher than that in the public monitoring system in a wide parameter space. Although the perfect monitoring system seems to achieve the highest level of cooperation, our results surprisingly reveal that the private monitoring system, despite being an imperfect information situation, can achieve a higher level than the public monitoring system.

The Pareto improvement of the private monitoring system is satisfied when the cost-benefit ratio of the giving game exceeds a threshold (Fig. 6) and the mutation rate is small (Fig. 7). The mutation rate, *μ*, has a great impact on the superiority of the private monitoring system over the public one. As shown in Supplementary Information, the private monitoring system achieves higher cooperation rates than the public system if the number of players is greater than a threshold, N*, where (10 < N* < 500) and the observation probability is greater than a threshold, q*, where (0.1% < q* < 1%), regardless of the degree of the two types of errors (*e*_1_ and *e*_2_).

## Discussion

Retributive justice or reciprocity alone can keep a cooperative regime under public information. However, the situation drastically changes by adopting imperfect monitoring systems. Changing the monitoring systems gives rise to a pertinent point on the influence of a system that does not guarantee a player’s single image. Stern-judging is evolutionarily stable in the public monitoring system yet not in the private monitoring system. Table 2 and Fig. S3(d,e) in the Supplementary Information show that the type of monitoring system (public or private) is essential for maintaining cooperative regimes in stern-judging (and shunning) regardless of the difference in the initial conditions, even if *q* = 1.

**Table 2:** The cooperative stable norms in public monitoring.

Norm	Stability	Cooperation rate
Image-scoring	75%	89.56%
Simple-standing	100%	94.19%
Stern-judging	100%	93.99%
Shunning	100%	55.50%
Staying	100%	94.14%

Details are the same as Table 1 except for *q* = 1.

This is because two discriminators may not correspond with a focal target in the private monitoring system, while they absolutely correspond in the public monitoring system. In the public monitoring system, even if an error in perception occurs, all the discriminators make a corresponding mistake, and thus, their assessment information absolutely corresponds. In contrast with the public monitoring system, the private monitoring system with perception errors does not always guarantee any concurrent image of a player in the eyes of all discriminators. When a game is played, most discriminators do not make mistakes in perception while the other discriminators mistake the image of a donor.

While every justified defection is absolutely justified in the public monitoring system, a justified defection in the private monitoring system is not necessarily justified. We call this the *justification dilemma* that emerges when a private monitoring system is assumed. That is, coercively uniformed assessment (public monitoring) allows that intolerant reciprocity drives a society to be stable. In contrast, private monitoring allows that intolerant reciprocity is not evolutionarily stable because the unconditional defectors can invade the reciprocity.

It is more likely that the tolerant norms overcome the justification dilemma than the strict norms. To understand the mechanism, we consider a specific situation. Assume that an image of a recipient in the eyes of an observer is *bad* while that in the eyes of the donor is *good* and that no error occurs. Note that both the donor and the observer are the discriminators. Following the action rule, the donor chooses cooperation (this is because the recipient is *good*). The observer assesses the donor as *bad* in the case of stern-judging and shunning, and as *good* in the case of staying and simple-standing. The difference has a considerable impact on the cooperative regime. In the case of the strict norms, justified cooperation by the donor is not justified and is a trigger to spread the *bad* reputation (this is because all of the observers assess the donor as *bad*). The strict norms mean that a *bad* reputation seldom changes to a *good* reputation. This mechanism shows that the cooperative regime finally collapses. Conversely, in the case of the tolerant norms, the justified cooperation is actually justified, and thus, the image of the donor is never damaged. Keeping a *good* reputation also maintains the cooperative regime.

Our simulation shows that staying is more likely to stabilize cooperative regimes than is simple-standing in a private monitoring system. This is because, as shown in Table 1, staying is easier to prevent from invading defectors than is simple-standing. Moreover, Fig. 4 shows that the basin of attraction in staying is wider than that in simple-standing. Figures 2(a and b) and 3(a and b) also support the advantage. Although both norms can make cooperation dominant in a regime even in a private monitoring system, a rigorous comparison shows the robustness of staying rather than of simple-standing. In a private monitoring system, the discriminators do not observe many games. In private monitoring, a rule that reserves assessments for any action to the *bad* players (staying) is more adaptive than a rule that absolutely assesses as *good* any action to the *bad* players (simple-standing). This result suggests that preserving assessment is an important factor in sustaining a cooperative society with less surveillance.

Our model reevaluates the role of unconditional cooperation, a naive strategy. Unconditional cooperators have often been assessed as detrimental under the image-scoring norm, because they are the so-called second-order free-riders who shirk paying the cost for excluding defectors through withholding help (that is, the justified defection). In most studies on the evolution of cooperation by indirect reciprocity with public monitoring, the key point is how to exclude such naive cooperators. In contrast, indirect reciprocity with private monitoring offers the unconditional cooperators a part of a solution. Under tolerant reciprocity, staying and simple-standing can maintain a cooperative regime jointly with unconditional cooperators. Further, the average payoff (the cooperation rate) of the cooperative stable point with private monitoring is higher than that of public monitoring if the cost-benefit ratio of the giving game is high and the mutation rate is low. This is because the unconditional cooperators play a role in boosting the cooperation rate of the population, while tolerant reciprocity protects against the invasion of the unconditional defectors^49, 50^.

We investigated an individual-based model in terms of evolutionary game theory, and its implications bear substantial discussions. The private monitoring system is a natural assumption compared with the perfect monitoring system. Our results suggest that the solidarity of retributive justice (discriminators) and philanthropism (unconditional cooperators) is important for contributive regimes with private monitoring systems, which indicates low surveillance levels. Enhancing surveillance and improving the degree of surveillance ousts the philanthropism or selfless contribution. The Panopticon^51^ proposed by Jeremy Bentham is like a lookout tower where a surveillance agent always monitors the prisoners. Bentham pessimistically predicted that such a perfect surveillance system would be a feature of modern society, and his prediction seems to be modificatorily realized in the advanced technological and information society^52–54^. The social welfare in modern society can be enhanced not by Panopticon, but by the rehabilitation of copybook maxims such as philanthropism.

## Methods

### Social norms

This section describes our model. We assume a finite population consisting of *N* players. We consider three strategies: unconditional cooperators who always cooperate (give help), unconditional defectors who never cooperate (withhold help), and discriminators. This paper considers five different norms for the discriminators: image-scoring, simple-standing, stern-judging, shunning and staying. The discriminators have private binary assessments (G: *good* or B: *bad*) for each player. The action rule of the discriminators is simple regardless of the norm type: cooperate (C) to those assessed as *good* and defect (D) to those assessed as *bad*. An assessment rule of the discriminators needs two types of information: the donor’s action (C/D) and the recipient’s image (G/B). All of the assessment rules of the five norms considered are given in Table 3.

**Table 3:** Assessment rules of the norms.

	Donor’s action (C/D) and Recipient’s image (G/B)			
	(C, G)	(D, G)	(C, B)	(D, B)
Image-scoring	G	B	G	B
Simple-standing	G	B	G	G
Stern-judging	G	B	B	G
Shunning	G	B	B	B
Staying	G	B	R	R

C: cooperate; D: defect; G: good B: bad; R: reservation (meaning that the discriminator keeps the donor’s image regardless of her or his action).

### Giving game and two error types

In each round, a donor and a recipient pair is randomly selected from the finite population. The donor plays a giving game and decides whether to cooperate with the recipient at fixed personal costs, *c* > 0. The recipient receives benefits, *b*, with *b* > *c* if and only if the donor cooperates. Self-interested players will contribute nothing because cooperators do not benefit from their own cooperation. Thus, our model—with no iteration of the interaction of the same players—reveals a social dilemma. Switching to defection improves the individual payoff, whatever the opponent’s action; however, this leads to mutual defection of payoff 0, which is worse off than mutual cooperation of payoff *b* − *c* > 0. All of the players face two error types: those in implementation and those in perception. The donor defects in contradiction to her or his intention to cooperate with a probability, *e*_1_, and the observer oppositely mistakes the assessment for the donor with a probability, *e*_2_.

### Private monitoring system

In a private monitoring system, we assume that each discriminator observes a game with the probability, *q*. In this situation, all of the discriminators have a chance to observe a game. Each potential observer rolls a dice. With the probability of *q*, the observer can observe a game, otherwise the observer cannot. If *q* = 1 and errors in perception never occur, then the situation is the same as in a public monitoring system.

### Individual-based model of evolution of reputation-based indirect reciprocity

To explore the evolutionary dynamics of a private monitoring system, we analyze the marginal value of a *good* reputation^38^. In the framework, any expected probability of a player’s image in the eyes of a discriminator is saturated if the games infinitely continue. We set it that sequential *T* rounds make a generation. When every generation begins, each discriminator rolls a dice and uniformly chooses an integer from a set of 0 to *N* and assigns the integer as the number of *good* players in the eyes of the discriminator. The *good* players are randomly assigned. The players never change their strategies through a generation, while the discriminators update their private assessments of the donors in every round if observed. To wait for the saturation of the player’s images, neither a benefit nor a cost occurs until *T*_*s*_ rounds in each generation, with *T*_*s*_ < *T*.

### Updating strategies

At the end of each generation, two different updating processes are performed. The first process is on learning. Every player has a chance to update her or his strategy and the frequency of *good* players. A player (set to *x*) randomly chooses a model player (set to *y*) among all of the players. The probability of the player changing her or his strategy and the frequency of *good* players is calculated as the following Fermi function^55^

$$P(x;y)=\frac{1}{1+{e}^{-\beta ({\pi }_{y}-{\pi }_{x})}},$$

where *π*_*x*_ is the average payoff of player *x*, *x*’s final payoff divided by the expected number of playing donors (recipients) after the saturation periods, *T*_*s*_. Then, player *x* decides whether to change her or his strategy and the frequency of *good* players. With the probability *P*(*x*; *y*), player *x* changes her or his strategy and the frequency of *good* players to player *y*’s, and otherwise, player *x* keeps her or his own strategy and the frequency of *good* players. The second updating process is on a mutation. A mutation occurs to keep the diversity of the strategies. Each player is replaced with a mutant player with a probability, *μ*. The strategy of the mutant player is randomly chosen from the three strategies: unconditional cooperator, unconditional defector, and discriminator.

### Calculating the basin of attraction

To calculate the basin of attraction in the individual-based simulation, we develop a method using Absorbing Markov Chain^56, 57^. We first calculate a transition matrix, *M*, of all of the points on the state space, *S*, and then calculate the absorbing probabilities, *B*. This method can be generally adopted if at least one attraction of the system is revealed.

The details are as follows. Let *N* be the number of player, *x*, *y*, *z* be non-negative integers, and the state space be defined as *S* = {(*x*, *y*, *z*)|*x* + *y* + *z* = *N*} = {(0, 0, *N*), (0, 1, *N* − 1), (0, 2, *N* − 2), …, (0, *N*, 0), (1, 0, *N* − 1), (1, 1, *N* − 2), …, (*N*, 0, 0)}. Each element of *S*, *s* = (*x*, *y*, *z*), denotes a playing population consisting of *x* cooperators, *y* defectors and *z* discriminators.

First, a transition on *s* is calculated by the simulation. Multiple trials are performed on the point *s*. In each trial, one generation only is performed and the number of each strategy in the next generation is counted after the updating process. The new distribution of strategies is denoted as *s*′ = (*x*′, *y*′, *z*′)∈*S* where *x*′, *y*′ and *z*′ are, respectively, the number of cooperators, defectors, and discriminators. By gathering pairs of (*s*, *s*′) for all of the trials on all of the points, the transition on *s* is defined. For example, *m*_*st*_ = 0.3 if three of ten trials on the point *s* go to the point *t*. ∑_*j*_ *m*_*ij*_ = 1 is satisfied. By simulating all of the points in *S*, the transition matrix, *M* = (*m*_*ij*_), a square matrix of dimension |*S*|, is calculated.

Next, the absorbing probabilities, *B* = (*b*_*s*_), a vector of dimension |*S*|, is calculated. *b*_*s*_ is denoted as the probability of reaching a cooperative regime, *S*_*c*_, from the point *s* ∈ *S* after infinite time steps. *S*_*c*_ is a set of {(*x*, 0, *z*)|*x* + *z* = *N*} if no mutation occurs. Instead, the non-cooperative regime, *S*_*d*_, is a set of {(0, *N*, 0)}. We set *b*_*s*_ = 1 when *s* ∈ *S*_*c*_ and *b*_*s*_ = 0 when *s* ∈ *S*_*d*_. The time independence means that, *B* = *MB* is satisfied when the system goes to a steady state, and thus, *B* can be calculated. The steady distribution can be regarded as the basin of attraction in the version of the individual-based simulation.

### Data availability

The simulation code is available in the supplementary information.

## Electronic supplementary material

Supplementary Information
