# Supplementary material for: Tolerant indirect reciprocity can boost social welfare through solidarity with unconditional cooperators in private monitoring
Source: Sci Rep. 2017 Aug 29;7:9737. doi: 10.1038/s41598-017-09935-2 (PMC5575281; doi:10.1038/s41598-017-09935-2)
Supplement: Supplementary file 1 — Supplementary Information [file 41598_2017_9935_MOESM1_ESM.doc]

**Supplementary Information for:**
**Tolerant indirect reciprocity can boost social welfare through solidarity with unconditional cooperators in private monitoring**

Isamu Okada (Soka), Tatsuya Sasaki (Vienna), Yutaka Nakai (Shibaura)

**Simulation run length**

Each run of our simulation basically carried 30 generations. Although each generation consists of 100,000 periods, the simulation run length may be considered rather low. We observed 100-generation runs for each norm with both monitoring systems. As shown in Figs. **S1** and **S2**, 30 generations are sufficient for some cases while even 100 generations are not enough for others. Note that the values of the cooperation rates differ even in the first generation because it depends on the saturated frequency of *good* players.

In the public monitoring system shown in Fig. **S1**, the robust cooperative regimes continue in all norms except for shunning and image-scoring. Shunning is not included in the leading eight^29^, and thus, even the public monitoring system does not keep cooperative regimes. This is consistent with Table **1b** in the main text, because shunning dominates in the population while the cooperative ratio is still vulnerable. The cooperation rates of image-scoring are highly flexible. The dynamic system has a neutral drift between unconditional cooperators and discriminators according to the theoretical analysis of the replicator dynamics of the system by Sasaki et al. (2017)^37^. The neutral drift effect makes the dynamic system fragile.

In the private monitoring system shown in Fig. **S2**, cooperation rates are basically fragile. In shunning, no trial can reach the cooperative regime as consistent with Table 1a. In stern-judging, the discriminator-dominated regime can be invaded by the unconditional defectors. Staying has a path to cooperative regimes of a co-existence of discriminators and unconditional cooperators. Simple-standing and image-scoring have two different paths towards cooperative regimes as well as staying and towards defective regimes dominated by unconditional defectors. Fig. **5** in the main text also shows that even staying has the second path to defective regimes.

The point of the difference relates to the stability of stabilizing points. On the one hand, in a homogenous stable point consisting of 100% defectors or 100% discriminators, a dominant norm has a significantly higher payoff than the other norms, and thus, it keeps regardless of the randomness in the simulations. On the other hand, in a stable point consisting of a mixture of multi norms, their payoffs are almost the same, and thus, the highly unstable turbulence due to randomness in the simulation is more likely to make the cooperative regimes collapse.

Because of a high degree of turbulence in our individual-based simulation, we adopt the version of 100 trials in 30 generations in the main text. This is because our focus is not on stability but on the cooperation rate of stable cooperative regimes. Instead of a rigorous stability check, Table **1** and Fig. **5** in the main text show the stability of cooperative regimes of each norm in the long term.

**Effect of degree of monitoring and two types of errors**

Fig. **S3** shows the difference of degree of monitoring (*q*) on the simulation results. The image-scoring norm does not use second-order information, and thus, observation probability does not influence the system. The stable mixture of the tolerant norms (staying and simple-standing) depends on the degree of monitoring: the weaker the monitoring system, the greater the fraction of the unconditional cooperators. A weak private monitoring system is likely to keep a cooperative regime with the unconditional cooperators, while a strong system is more likely to keep a cooperative regime with discriminators. This issue deserves further discussion.

Figs. **S4** and **S5** show the difference between two types of errors (*e_1_* and *e_2_*) on the simulation results. Although both types of errors (implementation errors and perception errors) slightly decrease cooperation rates in image-scoring, simple-standing, and staying, the degrees are almost the same.

**Advantage of the private monitoring system**

In this section, we deal with tolerant norms, staying and simple-standing, and compare the cooperation rates in the private monitoring system with those in the public monitoring system. Figs. **6** and **7** in the main text show that the private system takes an advantage over the public system when the cost-benefit ratio of the giving game is high and the mutation rate low. In this section, we check the influence of the other parameters: *N*, *q*, *e_1_*, and *e_2_*. As shown in Figs. **S6** to **S9**, neither *e_1_* nor *e_2_* has an influence on the advantage. Both *N* and *q* have a threshold to achieve the advantage.

**Staying and strict-standing**

Staying seems close to strict-standing (or L7 ^26^), a third-order social norm proposed in Ohtsuki and Iwasa (2004)^10^. In this section, we explain the difference. Staying does not use the image of the donor in making assessments of the image of the donor, and thus, according to the classification criteria^10^, staying is a second-order social norm. In contrast to staying, strict-standing, one of the leading eight^29^, uses the image of the donor in making assessments (e.g., accessing and updating a memory database of individual images). The difference between staying and the third-order social norms such as strict-standing is crucial when considering effects of load and error in perception and information transfer. In our model, the staying-norm adopter skips one’s assessment for a donor when one meets a bad recipient, and so, one absolutely keeps the assessment regardless of errors occurring in perception. Nonetheless, we emphasize that comparison of staying with strict-standing focusing on various aspects including ignorance, errors, cognitive costs, and experimental approach will be important in future works.

**Supplementary figures**


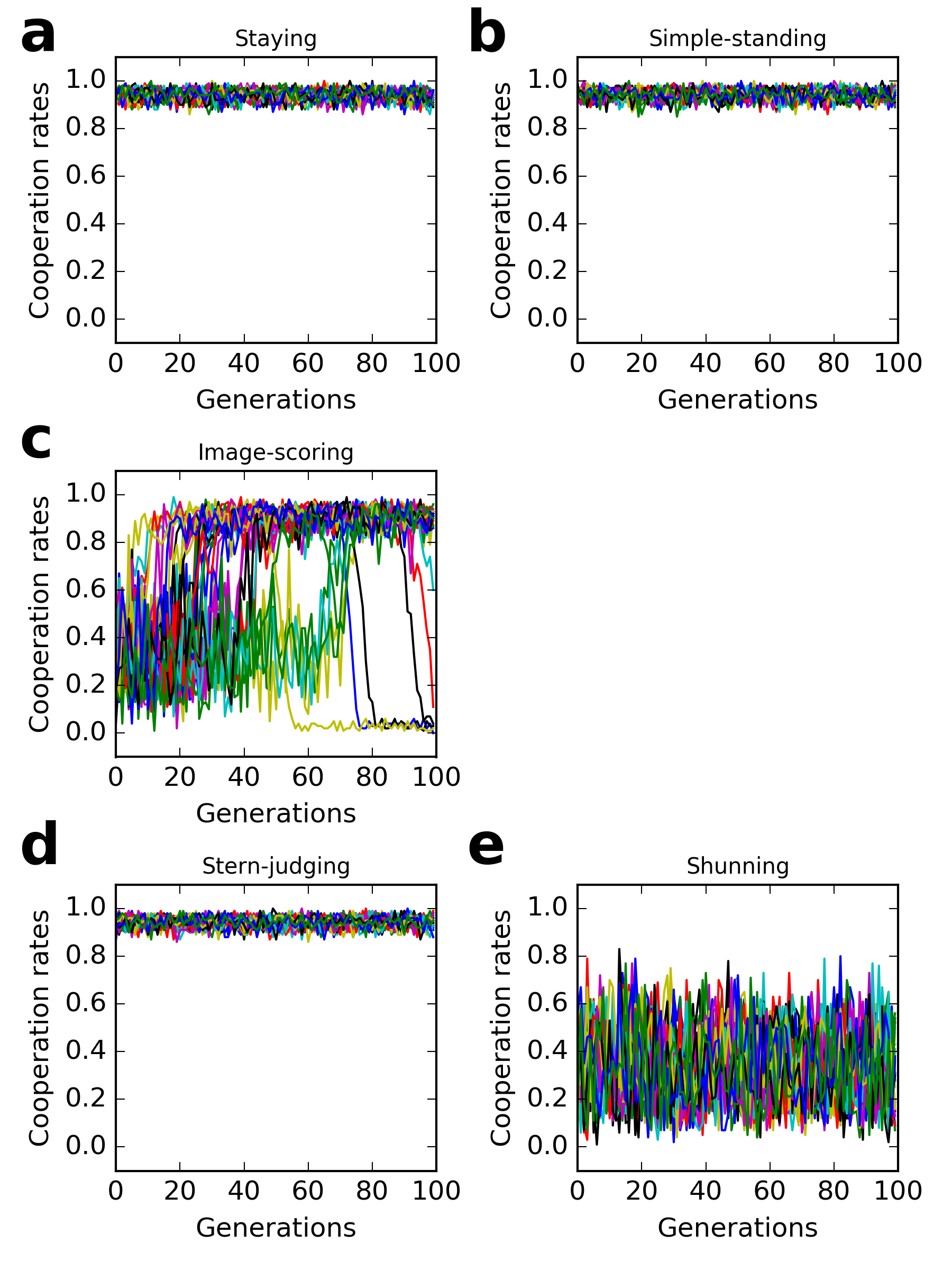


**Fig. S1.** **Cooperation rates in public monitoring systems with 30 trials.** Each line reflects time series data of a trial of 100 generations in **a** staying, **b** simple-standing, **c** image-scoring, **d** stern-judging, and **e** shunning. An initial population consists of 100% discriminators. The parameter values are the same as Table 1 in the main text.

**
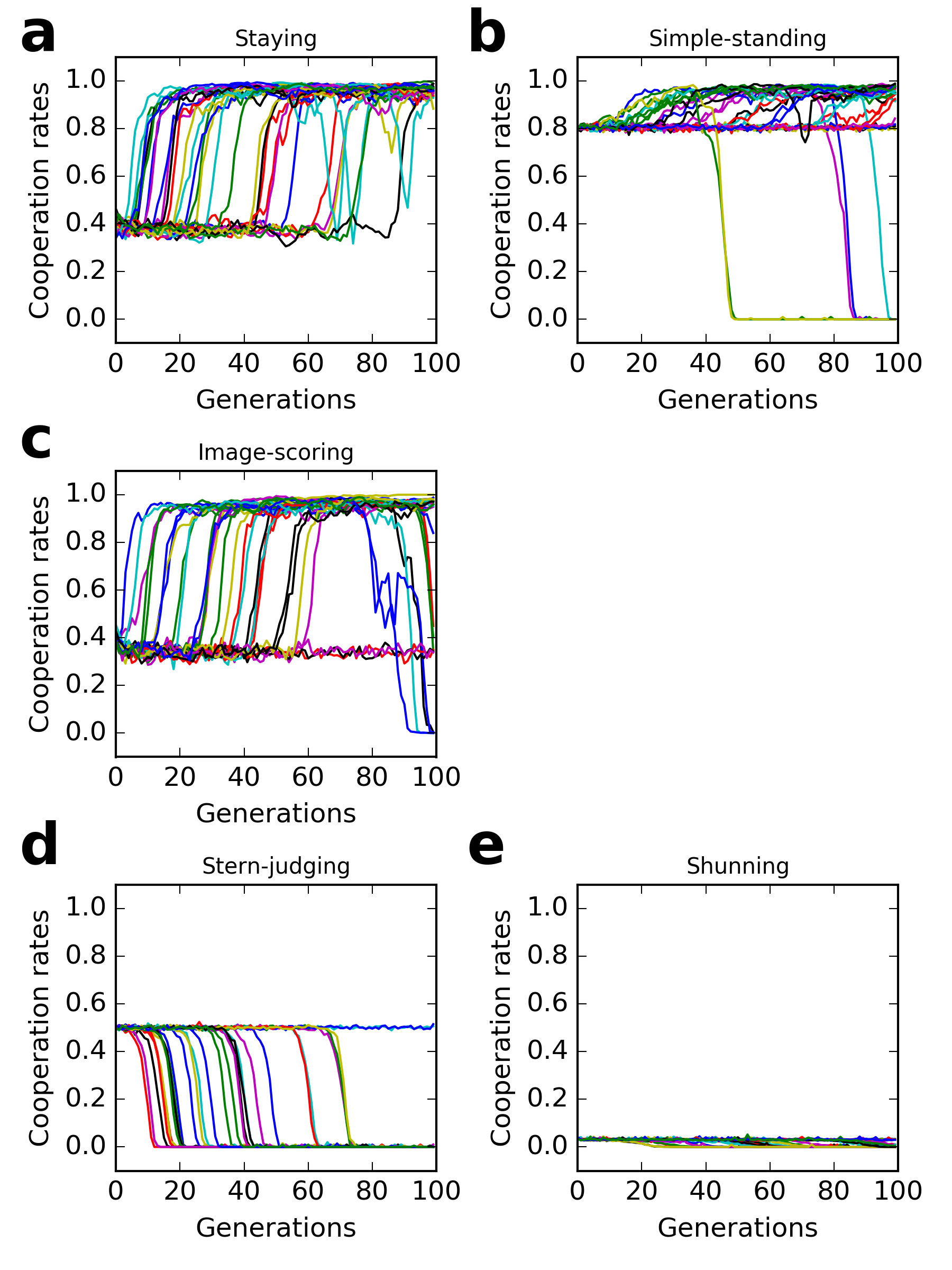
**

**Fig. S2. Cooperation rates in private monitoring systems with 30 trials.** Details are the same as Fig. S1.

**
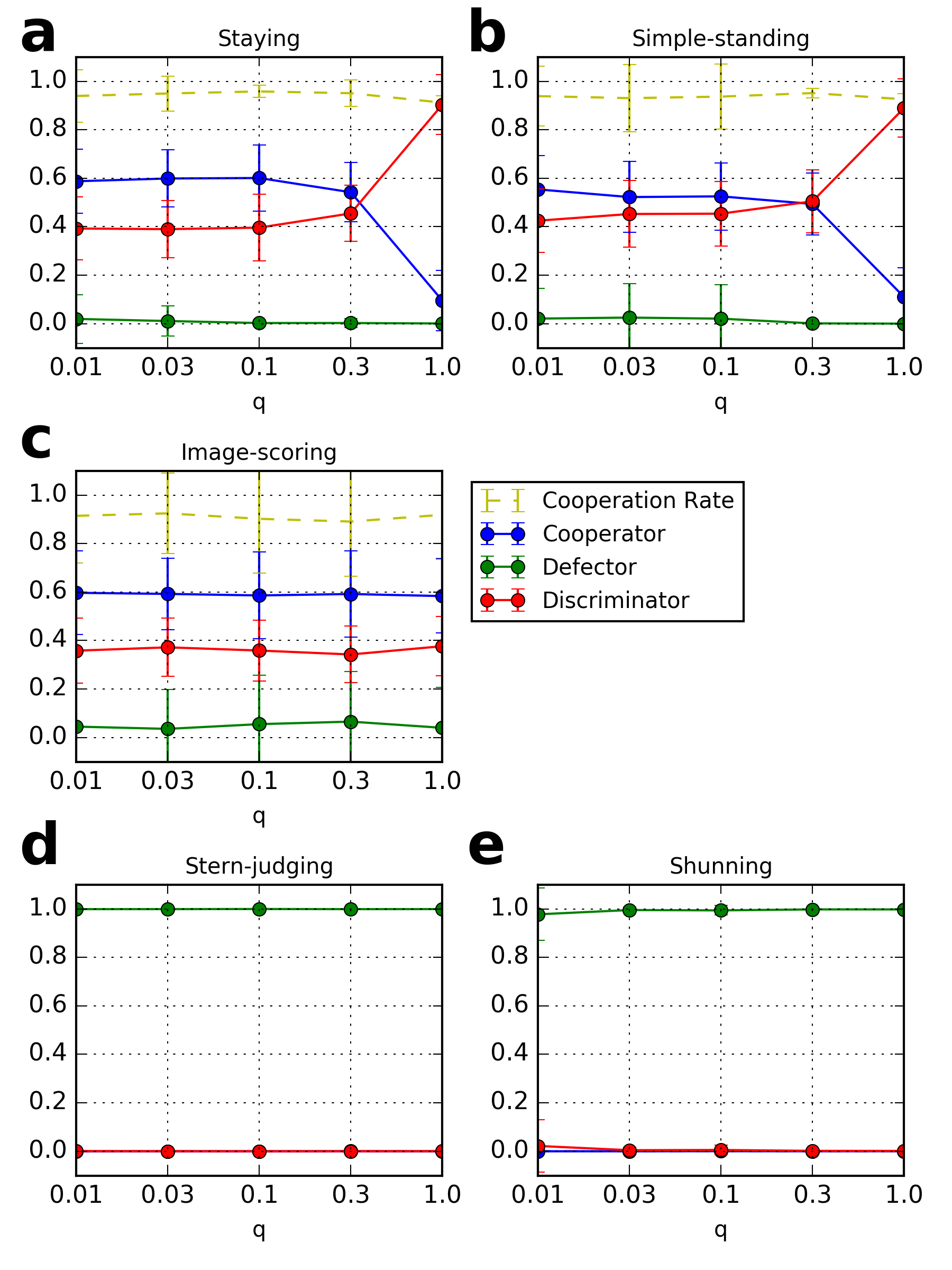
**

**Fig. S3. Cooperation rates and strategies in a private monitoring system with different degrees of monitoring, *q*.** The parameter values are the same as Fig. 2 in the main text except for *b=3* and *q* (a variable).


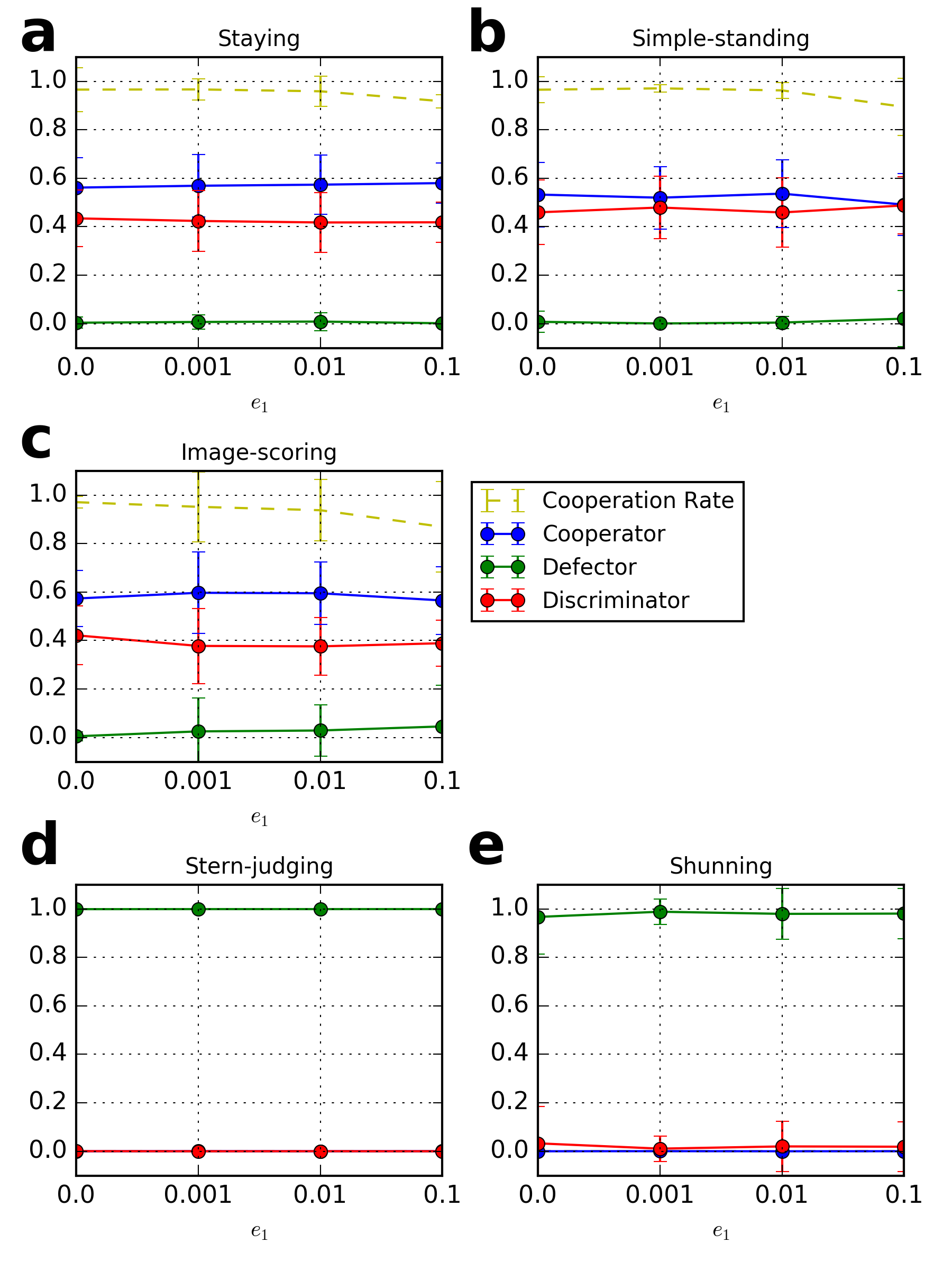


**Fig. S4. Cooperation rates and strategies in a private monitoring system with different action error, *e_1_*.** The parameter values are the same as Fig. 2 in the main text except for *b=3* and *e_1_* (a variable).


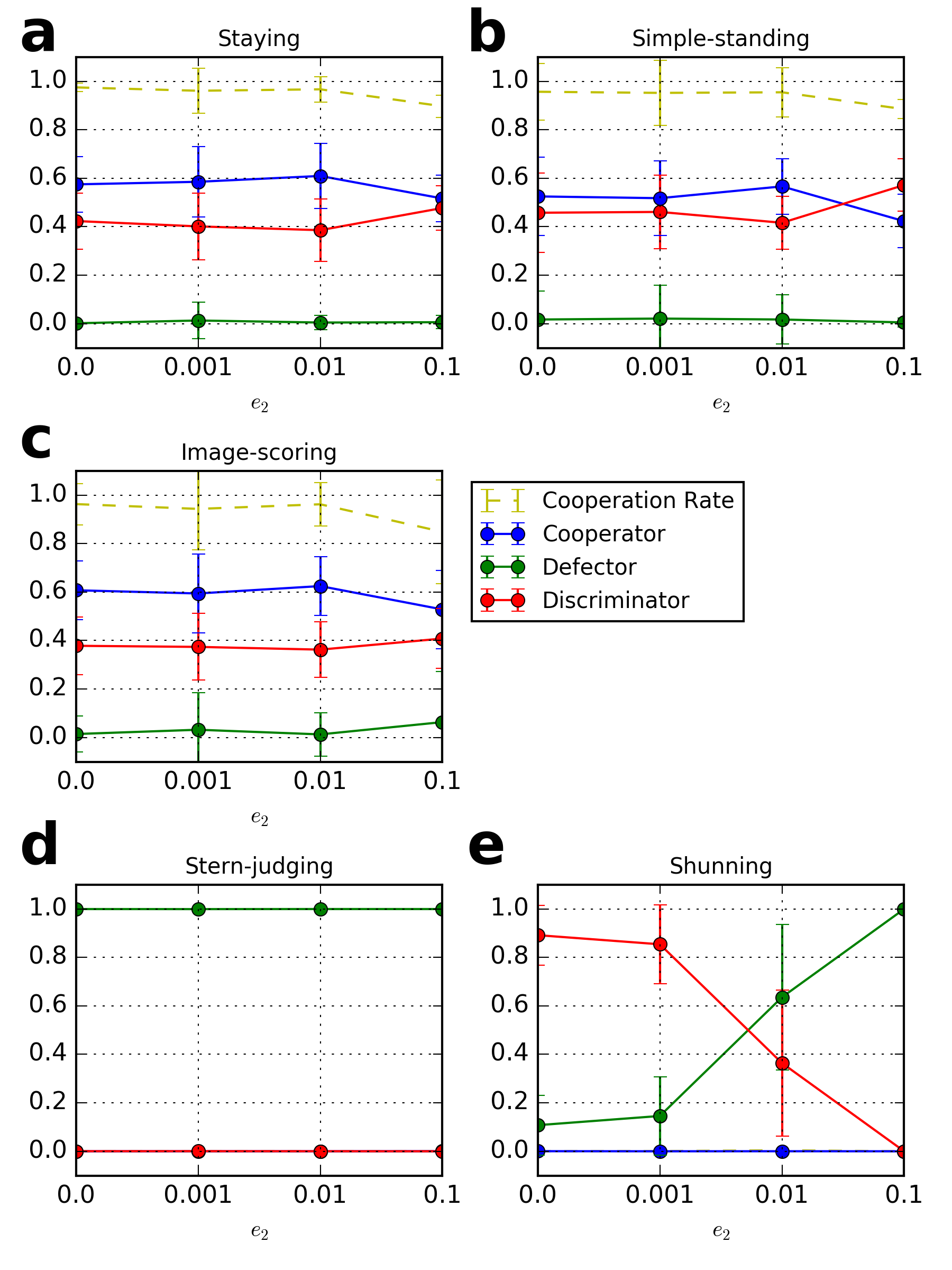


**Fig. S5. Cooperation rates and strategies in a private monitoring system with different perception error, *e_2_*.** The parameter values are the same as Fig. 2 in the main text except for *b=3* and *e_2_* (a variable).


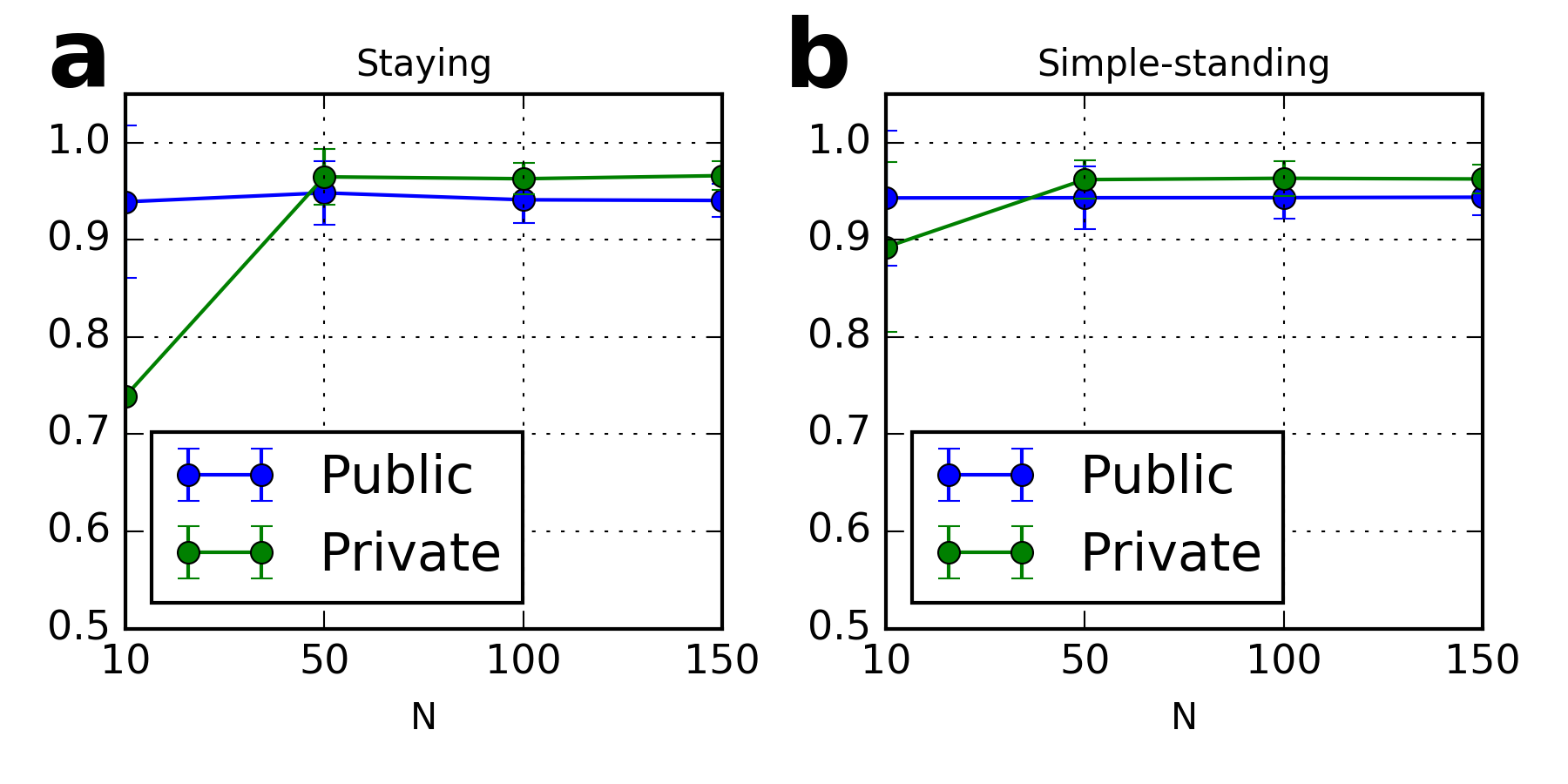


**Fig. S6: Cooperation rates of stable states with different number of players, *N*.** **a** staying and **b** simple-standing. The cooperation rate with private monitoring exceeds that with public monitoring if *N* >10 in both **a** and **b**. The simulation settings are the same as Fig. 6 in the main text except for *b*=3 and *N* (a variable).


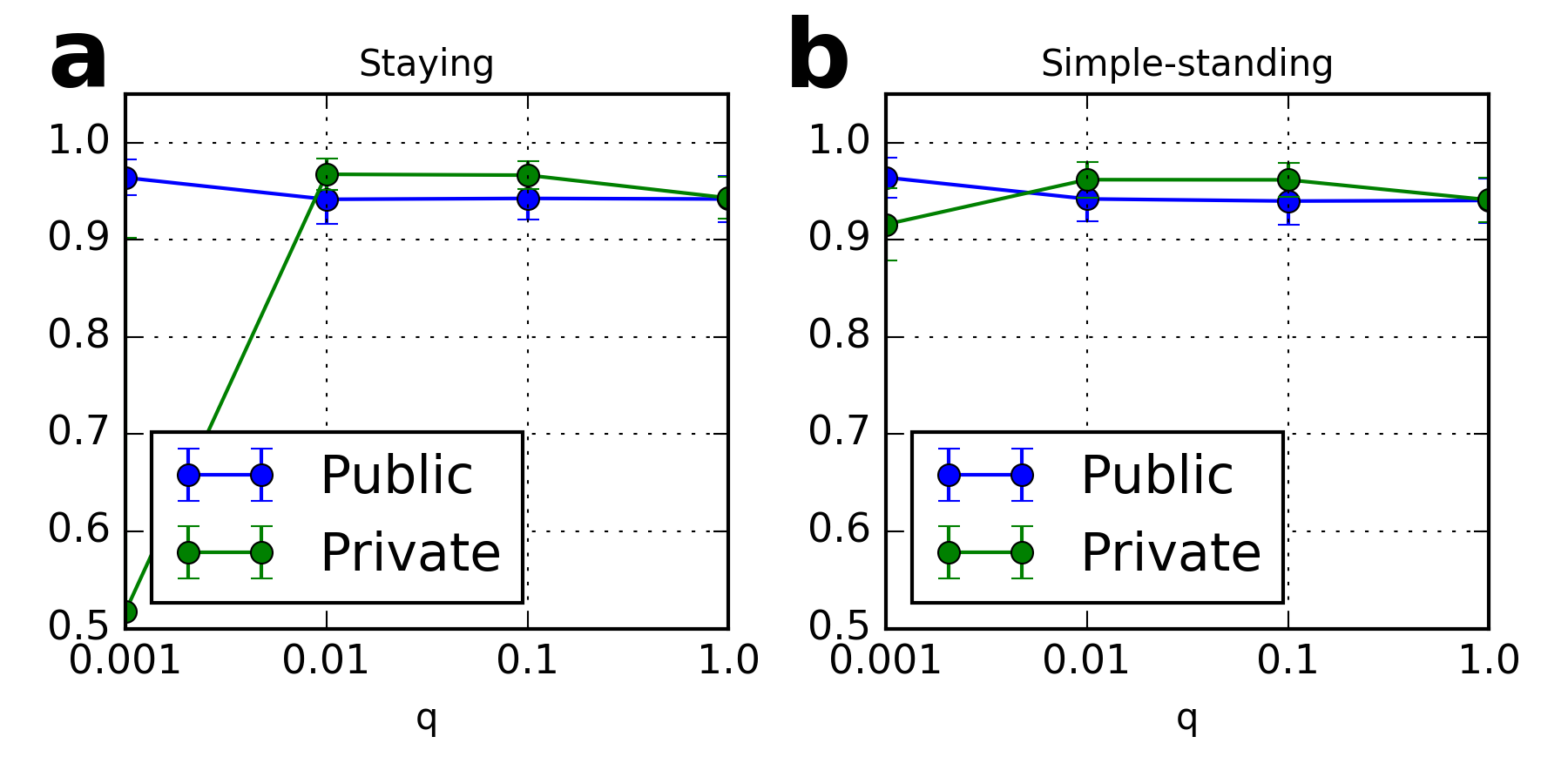


**Fig. S7. Cooperation rates of stable states with different observation probability, *q*.** **a** staying and **b** simple-standing. The cooperation rate with private monitoring exceeds that with public monitoring if *q* >0.001 in both **a** and **b**. The simulation settings are the same as Fig. 6 except for *b*=3 and *q* (a variable).


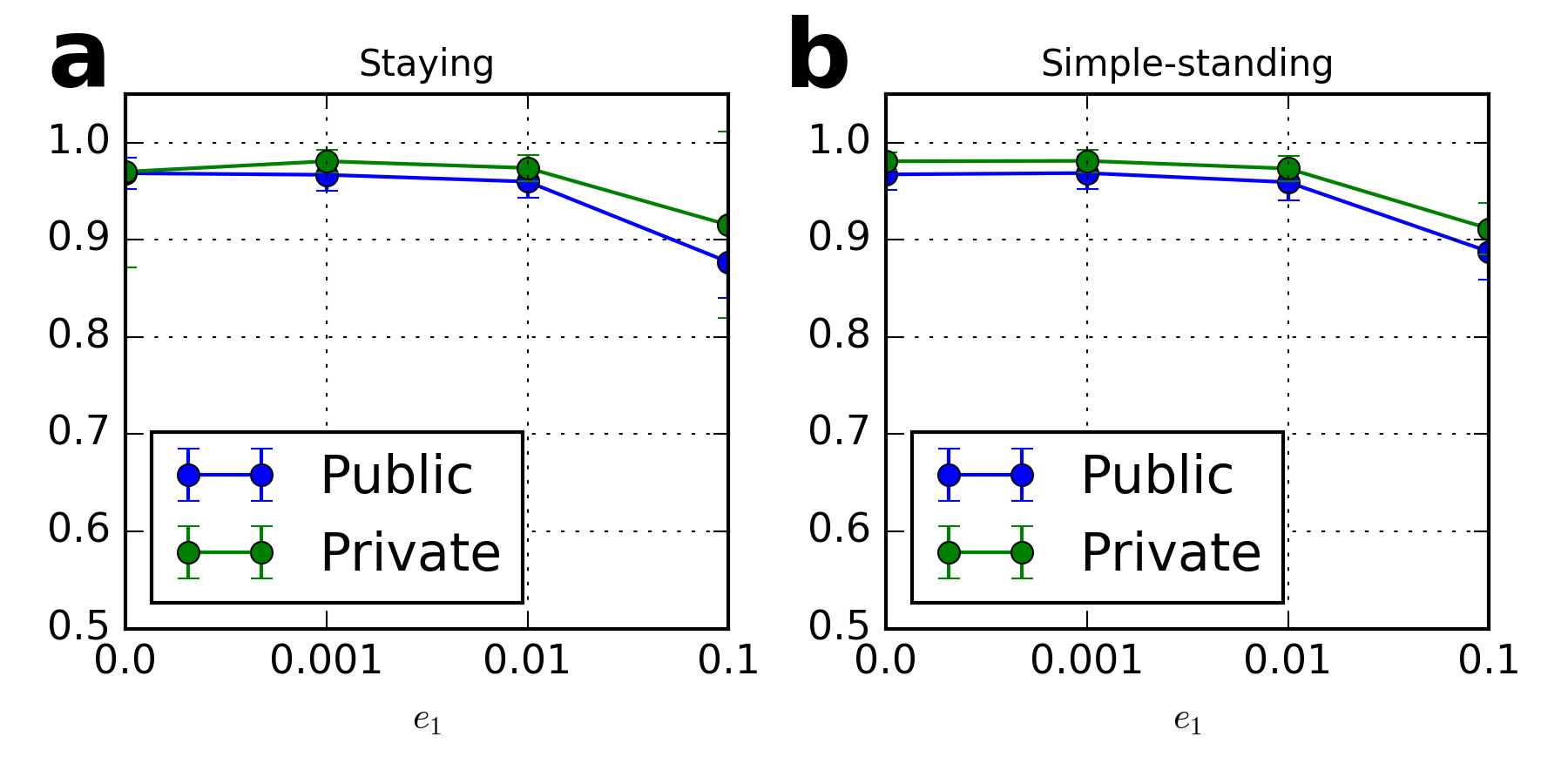


**Fig. S8. Cooperation rates of stable states with different action error, *e_1_*.** **a** staying and **b** simple-standing. The cooperation rate with private monitoring exceeds that with public monitoring regardless of *e_1_* in both **a** and **b**. The simulation settings are the same as Fig. 6 in the main text except for *b*=3 and *e_1_* (a variable).


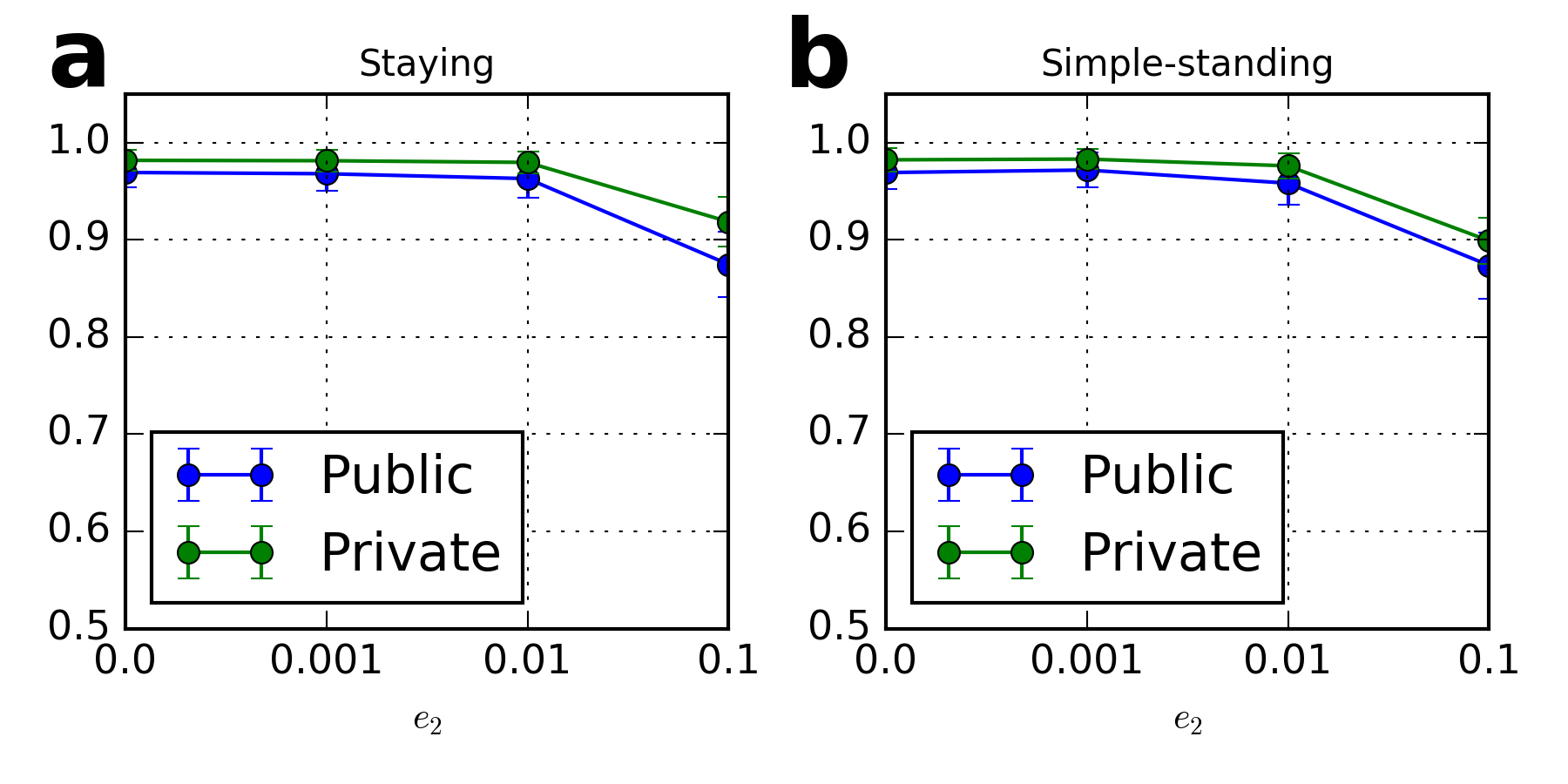


**Figure S9. Cooperation rates of stable states with different perception error, *e_2_*.** **a** staying and **b** simple-standing. The cooperation rate with private monitoring exceeds that with public monitoring regardless of *e_2_* in both **a** and **b**. The simulation settings are the same as Fig. 6 in the main text except for *b*=3 and *e_2_* (a variable).

**Python code of the model**

# A code of Python for the paper titled

# 'Tolerant indirect reciprocity can boost social welfare through solidarity with unconditional cooperators in private monitoring'

# made by Isamu Okada at July 3rd, 2017

import numpy as np

import pylab as p

import random as r

class Global():

const = {

'Scenario':100, # Trials

'Gnr' : 30, # Generation

'Prd' :100000, # T (Periods)

'Avail': 90000, # Ts

'N' : 100, # Players

'Fermi': 3.0, # Coef. Learning

'mu' : 0.001, # Mutation rate

'q' : 0.01, # Observation prob.

'b' : 3.0, # benefit

'c' : 1.0, # cost

'e1' : 0.03, # Implementation errors for C -> D

'e2' : 0.03 # Asseessment errors

}

class Agent():

def __init__(self,id,type):

self.id = id

self.type = type[0]

ratio = type[1]

self.f = []

for i in range(Global.const['N']):

if i < ratio:

self.f.append(1)

else:

self.f.append(0)

r.shuffle(self.f)

self.payoff = 0.

def getType(self):

return self.type

def getId(self):

return self.id

def getRep(self,id):

return self.f[id]

def calRatio(self):

return sum([x for x in self.f])

def cost(self):

self.payoff -= Global.const['c']

def benefit(self):

self.payoff += Global.const['b']

def GupdateStaying(self,donor,recipient,act):

if self.f[recipient.getId()] == 1:

tag = act

if r.random() < Global.const['e2']:

self.f[donor.getId()] = 1 - tag

else:

self.f[donor.getId()] = tag

def GupdateStanding(self,donor,recipient,act):

if self.f[recipient.getId()] == 1 and act == 0:

tag = 0

else:

tag = 1

if r.random() < Global.const['e2']:

self.f[donor.getId()] = 1 - tag

else:

self.f[donor.getId()] = tag

def GupdateJudging(self,donor,recipient,act):

if self.f[recipient.getId()] == act:

tag = 1

else:

tag = 0

if r.random() < Global.const['e2']:

self.f[donor.getId()] = 1 - tag

else:

self.f[donor.getId()] = tag

def GupdateScoring(self,donor,recipient,act):

tag = act

if r.random() < Global.const['e2']:

self.f[donor.getId()] = 1 - tag

else:

self.f[donor.getId()] = tag

def GupdateShunning(self,donor,recipient,act):

if self.f[recipient.getId()] == 1 and act == 1:

tag = 1

else:

tag = 0

if r.random() < Global.const['e2']:

self.f[donor.getId()] = 1 - tag

else:

self.f[donor.getId()] = tag

def actionF(donor,recipient):

type = donor.getType()

if type == 0:

intend = 1

elif type == 1:

intend = 0

else:

intend = donor.getRep(recipient.getId())

if r.random() < Global.const['e1']:

intend = 0 # 1 - intend

return intend

def trajectory(Types,norm):

N = Global.const['N']

avail = Global.const['Avail']

q = Global.const['q']

A = []

discriminators = []

id = 0

for t in Types:

a = Agent(id,t)

A.append(a)

if t[0] == 2:

discriminators.append(a)

id += 1

for period in range(Global.const['Prd']):

players = r.sample(A, 2)

donor = players[0]

recipient = players[1]

act = actionF(donor,recipient)

if period > avail:

if act == 1:

donor.cost()

recipient.benefit()

candidate = discriminators[:]

if donor.getType() == 2:

candidate.remove(donor)

if recipient.getType() == 2:

candidate.remove(recipient)

for observer in candidate:

if r.random() < q:

if norm == 'Staying':

observer.GupdateStaying(donor,recipient,act)

elif norm == 'Standing':

observer.GupdateStanding(donor,recipient,act)

elif norm == 'Judging':

observer.GupdateJudging(donor,recipient,act)

elif norm == 'Scoring':

observer.GupdateScoring(donor,recipient,act)

elif norm == 'Shunning':

observer.GupdateShunning(donor,recipient,act)

return A

def fermi(a,b):

s = Global.const['Fermi']

div = (Global.const['Prd'] - Global.const['Avail'] )/ Global.const['N']

p = 1./(1. + np.exp(-1. * s * (b.payoff - a.payoff) / div))

if r.random() < p:

return [b.type,b.calRatio()]

else:

return [a.type,a.calRatio()]

def makeType(type):

N = Global.const['N']

if type == 0:

return [0,N]

elif type == 1:

return [1,0]

else:

return [2,r.randint(0,N)]

def obsF(A):

N = float(Global.const['N'])

N2 = float(N * N)

Prd = float(Global.const['Prd'] - Global.const['Avail'])

v = [0.,0.,0.]

sum = fsum = 0.

for a in A:

v[a.type] += 1

sum += a.payoff

fsum += a.calRatio()

for i in range(3):

v[i] /= N

sum /= Prd

fsum /= N2

return [v,sum,fsum]

def RD(norm):

N = Global.const['N']

mu = Global.const['mu']

v=[10,10,N-20]

Types = []

for i in range(N):

if i < v[0]:

Types.append(makeType(0))

elif i < v[0]+v[1]:

Types.append(makeType(1))

else:

Types.append(makeType(2))

r.shuffle(Types)

for time in range(Global.const['Gnr']):

print time

A = trajectory(Types,norm)

Types = []

for x in A:

if r.random() < mu:

Types.append(makeType(r.randint(0,2)))

else:

y = r.choice(A)

Types.append(fermi(x,y))

return obsF(A)

###############################################################

if __name__ == "__main__":

Norm = ['Staying','Standing','Judging','Scoring','Shunning']

for norm in Norm:

output = []

for scenario in range(Global.const['Scenario']):

print norm,scenario

output.append(RD(norm))

fp = open(norm + '.csv','w')

linex = '# Payoff, CRatio, X, Y, Z\n'

for v,s,c in output:

linex += str(s) + ',' + str(c) + ',' + str(v[0]) + ',' + str(v[1]) + ',' + str(v[2]) + '\n'

fp.write(linex)

fp.close
